# Editorial: The 4th dimension of the 3d chromatin organization: Dynamics and functional consequences

**DOI:** 10.3389/fcell.2022.1065753

**Published:** 2022-10-25

**Authors:** Anastassiia Vertii, Maria Ivshina, Shashi Kant, Andre J. van Wijnen

**Affiliations:** ^1^ Molecular Cell and Cancer Biology Department, UMASS Chan Medical School, Worcester, MA, United States; ^2^ UMASS Chan Medical School, Program in Molecular Medicine, Worcester, MA, United States; ^3^ Cardiovascular Division, Department of Medicine, Brigham and Women’s Hospital, Boston, MA, United States; ^4^ Department of Biochemistry, University of Vermont, Burlington, VT, United States

**Keywords:** chromatin dynamics, Stress, signaling, circadian rhythms, single-molecule

Chromatin organization is a function of time, regardless of the biological processes. Development, circadian rhythms, and cellular and environmental stress each influence chromatin architecture. The thriving development of technology to study the temporal 3D chromatin organization with time as its 4th dimension is facilitated by the multiple collaborative efforts in the field generating scientific knowledge hubs dedicated to temporal and spatial chromatin organization within the nucleus. For example, the 4DNucleome consortium (https://www.4dnucleome.org/) provides a major technological intersection that combines mapping, model simulation, and functional validation of chromatin dynamics. The current collection of articles in this special issue appreciates the cutting-edge technologies to study chromatin dynamics. A comprehensive article from Anna Chanou and Stephan Hamperl elegantly highlights single-molecule methods to study the positioning of nucleosomes, the basic unit of chromatin organization, DNA replication, transcription, and chromatin modifications. The authors discuss advances in the development of third-generation sequencing, force spectroscopy, imaging, and methylation footprinting (SAMOSA) - current efforts to address the fundamental challenges in studying chromatin dynamics, such as averaging parameters within a population of molecules or cells.

Tissue development in multi-cellular eukaryotes are mediated by intricate regulatory mechanisms that rely on chromatin dynamics. The combined analysis of epigenetic marks and transcriptomes is essential to comprehend the functional consequences of chromatin dynamics during development. The study by Baohua Tan et al. and co-authors in our collection provides a genome-wide overview of the facultative heterochromatin mark, H3K27me3, in relation to transcriptome analysis during porcine muscle development. This paper revealed a progressive increase in H3K27me3 during skeletal muscle development. Intriguingly, H3K27me3 also decorates promoters of differentiation-specific genes in porcine satellite cells, thus halting differentiation and preserving the pool for potential muscle regeneration.

Combined omics analysis is instrumental for our understanding of chromatin dynamics and functional outcomes during differentiation. The manuscript by Junhong Wang and colleagues in this collection provides an excellent example of how the combined application of using Hi-C, ATAC-seq, and RNA-seq methods yield major insights into the process of T cell differentiation. This study examined the role of a zinc finger and BTB containing 1 protein (Zbtb1), one of the key players in the immune compartment, which regulates T, B, and NK cell development. The combined omics analysis presented in this study revealed a novel role of Zbtb1 in modulating the T lymphoblast chromatin landscape.

The impact of the ecological macro-environment on epigenetic regulation is a fascinating facet of 4D chromatin organizations. This important topic was tackled in the research article by Shahrbanou Hossein and co-authors within our topic collection. Their study focuses on the epigenetic regulation of phenotypic sexual plasticity in zebrafish as a versatile biological model organism in which genetically defined sex that can be influenced by environmental factors. A skewed sex ratio might negatively affect population development during rapid environmental changes. This evolutionary process motivated the authors to investigate the regulatory role of epigenetic control in the process. The authors identified increased DNA methylation in male-biased families compared to female-biased families, as reflected by a higher number of methylated positions in testes *versus* ovaries. Environmental factors such as higher incubation temperatures resulted in higher DNA methylation, providing new insights into sex plasticity in response to the environment.

The intriguing intersection between environmental and physiological processes that influence genome organization is exemplified by studies on circadian rhythms. This topic is discussed in the review by Tartour and Padmanabhan. The authors discuss how circadian TADs (topologically associated domains) shift from active euchromatin regions (compartment A in Hi-C experiments) to inactive heterochromatin regions (compartment B) during transitions from active to repressive phases of the circadian rhythm. Current findings in the field indicate a striking “breathing” of chromatin domains on the time scale of hours depending on cellular demands for gene expression. During the “day-night” shift, the intra-TADs also undergo dynamic rearrangements at the level of promoter-enhancer loop organization. Compartment B-targeted circadian TADs are localized at the nuclear periphery, which represents one of the two principal locations for heterochromatin domains (i.e., lamina-associated domains, LADs) that undergo dynamic alterations during cellular differentiation (Peric-Hupkes et al., 2010). Notably, there is no data regarding the circadian TADs in nucleoli-associated domains, NADs—the other major heterochromatin hub within the nuclei.

Inflammation and infection create severe stress on our bodies. The impact of stress factors (inflammatory cytokines, fever, viruses, and others) on chromatin organization remains *terra incognita*. The mini-review article by Anastassiia Vertii discusses the current state and challenges in stress-mediated alterations of the 3D chromatin organization. A significant amount of data suggests that stress influences chromatin organization at the level of loops, TADs, nuclear bodies, and chromosome territories, as was reported (Le et al., 2016). The extent to which effects are cell-type or tissue-specific remains to be established. Other key questions that remain to be addressed focus on the similarities and differences in the mechanisms that mediate stress-induced chromatin alterations compared to development. For example, inflammatory stimuli often represent cues for the differentiation of immune cells, thus providing a paradigm for studying both processes. The review paper also emphasizes the importance of organismal stress during post-natal development and homeostasis, where inflammatory insults that underlie multiple chronic disorders may provoke chromatin remodeling during both inflammatory and recovery stages of tissue healing.

In summary, the topic collection we have procured to illustrate the 4^th^ dimension of 3D chromatin organization covers aspects of chromatin dynamics, including current methodologies, organismal and tissue development, the impact of circadian rhythms, environmental factors, and inflammatory stresses on chromatin dynamics. These topics represent critical directions that exemplify the multifactorial influence of time on the chromatin landscape. An impressive suite of technologies, such as single-molecule and single-cell omics, allows for investigations of chromatin dynamics with unprecedented detail and addresses the pervasive problem of population averaging. Although current progress is cause for optimism, the integration of the complex omics data sets obtained with different methods across multiple time-dependent biological processes remains a formidable task.
